# Effect of Novel Rhythmic Physical Activities on Fundamental Movement Skills in 3- to 5-Year-Old Children

**DOI:** 10.1155/2020/8861379

**Published:** 2020-12-28

**Authors:** Xin Hu, Gui-ping Jiang, Zhong-qiu Ji, Bo Pang, John Liu

**Affiliations:** ^1^College of Physical Education and Sports, Beijing Normal University, Beijing 100875, China; ^2^Department of Physical Education and Health Education School of Health, Springfield College, 01109, USA

## Abstract

Based on traditional rhythmic physical activities (TRPA), we created novel rhythmic physical activities (NRPA). The purpose of this study was to verify the effectiveness of NRPA in improving children's fundamental movement skills (FMS). 289 children (135 boys, 154 girls) from 3 to 5 years old were randomly divided into an experimental group and a control group. Tools of Test of Gross Motor Development-2, Tekscan instrument, and balance beam were to assess children's FMS. Two-way repeated measures ANOVA was used to analyze and compare the changes in the related parameters of locomotor, manipulative, and balance movement skills before and after intervention (groups × time). NRPA performed significantly better than TRPA from pre- to posttest for FMS. Furthermore, NRPA had significantly higher posttest scores than TRPA. Therefore, NRPA could effectively promote the development of children's FMS due to the concept of the sensitive period of motor development, the variability of movement parameters, and the incomplete repeatability of practice methods.

## 1. Introduction

Fundamental movement skills (FMS) include locomotor, manipulative, and balance [1], which are considered to be the building blocks that lead to specialized motor sequences required for adequate participation in many organized and nonorganized physical activities for children, adolescents, and adults [2]. In recent years, there has been increasing research interest on the topic of FMS development as it relates to children's physical health [3] and cognitive and social development [4], as well as to provide the foundation for an active lifestyle [5, 6]. The interactions of FMS with perceptions of motor development and health fitness have been used to predict subsequent obesity from childhood to adulthood [6, 7].

FMS must be instructed and practiced; they do not develop automatically over time [2]. From a dynamic systems theoretical perspective, motor skill development is dynamic and based upon the interaction between constraints from the task, the learner, and the environment [8]. There is an emerging literature base to show the positive effects of early -motor skill programs on motor skill development for young children [9]. FMS are featured as a key part of the National Association for Sport and Physical Education (NASPE) in America [10] and Australia's Health and Physical Education [11]. In addition, this age period of 3 to 5 years old is a sensitive period [6, 12]; therefore, early exercise interventions for FMS acquisition are needed.

Rhythmic physical activities (RPAs) are defined as children's activities performed with the rhythm of music and have been widely implemented in kindergartens throughout China. Studies [13, 14] indicate that the rhythm of body movement and music has a close inner connection, and the sensitivity of the body's reaction to the rhythm of music determines the depth of human perception of music. RPAs integrate the natural tendencies of children with music and dance. Jaques-Dalcroze [15] founded eurhythmics teaching methods according to this component of human nature, which allowed children to experience the speed and strength of music and express their inner feelings through body movement. Based on the traditional rhythmic physical activities (TRPA) currently being carried out in Chinese kindergartens, we created novel rhythmic physical activities (NRPA) that incorporate the concept of motor development. The current study sought to explore the value of NRPA on the development of children's FMS, find a basis for the promotion of NRPA in kindergartens in China, thereby enriching the physical activity curriculum to develop FMS.

## 2. Materials and Methods

### 2.1. Participants

In this study, 289 children (135 boys, 154 girls) from 3 to 5 years old were selected from a total of eight classes in public kindergarten in Beijing to take the TGMD-2 (Test of Gross Motor Development-2), which were randomly divided into an experimental group and a control group. Then, 30 children were randomly selected from the experimental group and the control group for static and dynamic balance tests using Tekscan instrument and balance beam. Basic information about the physical conditions of the participants is shown in Table 1. Before running the test, the physician provided a detailed explanation of the research to the parents of the participants and then obtained their consent. In this study, the 3-year-old group included children between 3 and 4 years old who came from the bottom class in kindergarten, the 4-year-old group included children between 4 and 5 years old who came from the middle class in kindergarten, and the 5-year-old group included children between 5 and 6 years old who came from the top class in kindergarten.

### 2.2. Apparatus and Procedures

#### 2.2.1. TGMD-2

TGMD-2 was used to assess the children's gross motor skills; the test consists of two subtests: one assessing the performance of locomotion skills including running, jumping, hopping, leaping, sliding, and galloping; the other assessing manipulative skills including throwing, catching, kicking, striking, dribbling, and underhand rolling [16]. Verbal instructions and demonstrations were given following test guidelines. Each motor skill had 3 to 5 criteria, and a score of “1” was given for reaching the standard, otherwise 0. Each motor skill was performed twice, and the best score was taken. Then, the score was totaled. The final scores of locomotor and manipulative were analyzed. Each participant was given a practice trial followed by two consecutive tests that were used for analysis. All tests were videotaped in the sagittal view to allow for later assessment of interrater reliability. The TGMD-2 has been indicated to have good reliability and validity in many countries [17], including China [18]. Before the test, the two testers independently assessed the performance of 8 children (25% of the total sample) for the interrater reliability assessment. The Pearson correlation coefficients of the 12 motor skills were between 0.52 and 0.86, indicating that the correlations were statistically significant (*P* < 0.01). After confirming interrater reliability, each of the two testers assessed half of the group of children. A sample of 15 children (5 from each age group) was retested for the test-retest reliability analyses within 14 days after the intervention; the retest rate was 25%. The test-retest reliability was calculated to confirm the consistency of the TGMD-2 items and provide information about the scale's temporal stability. The Pearson correlation coefficient of the two test was 0.93, which was statistically significant (*P* < 0.01). Therefore, the TGMD-2 used in this study demonstrated good reliability.

#### 2.2.2. Test of Static Balance

A Tekscan foot-pressure measurement system (Foot Research 6.40 software, USA) was used to test the participants' static balance ability, while the participants were standing on both feet with eyes open (FEO), standing on both feet with eyes closed (FEC), and standing on a single foot, the dominant foot, with eyes open (SFEO). First, the participants performed 10 minutes of warm-up activities according to other researchers' static balance ability tests [19]. These activities mainly included jogging and aerobics, and then, three kinds of standing tests were performed in a room, with a duration of 10 seconds for each test. During the SFEO test, the physician put a soccer ball in front of the participant, told him or her to play soccer, and observed which foot the participant used to play soccer. The foot that the participant used to play the ball was considered the dominant foot. Participants were asked to remove their shoes, stand on a central location of the force plate, hang their arms naturally on both sides of the trunk, point their toes forward on the three tests, and direct their eyes to look forward. The acquisition frequency was 50 Hz, and in young children, the Tekscan foot pressure measure had good reliability and validity, which were 0.69 and 0.99, respectively [19–22].

In view of the laboratory equipment and the evaluation index values frequently used to evaluate children's static balance ability [19, 23, 24], achieving postural balance relies on the ability of the central nervous system to control the body's center of mass within the base of support [25]. This study selected, collected, and analyzed the envelope area (area), path length (length), and maximum displacement in the anteroposterior (A-P) and mediolateral (M-L) directions of the center of pressure (COP) to assess children's static balance performance [23]. The COP area is quantified as the largest area enclosed by the trajectory of the human body's center of gravity sampled over time, and the COP length is quantified as the total length of the trajectory of the center of gravity sampled over time; a large value corresponds to a poor balance ability. The maximum displacement of the COP in the A-P and M-L directions represents the degree of shaking, which is inversely proportional to the degree of balance ability [26, 27].

#### 2.2.3. Test of Dynamic Balance

Walking on a balance beam has often been used to test children's dynamic balance ability in China; therefore, we used this method to assess children's dynamic balance ability. A balance beam (3 meters long, 10 cm wide, 30 cm high) and a stopwatch were used to conduct the test. The two ends of the balance beam were the start and end lines, and each end had a platform (20 cm long, 20 cm wide, 30 cm high). Subjects performed moderate warm-up exercises before the test and then stood on the platform, facing the balance beam, with their arms extended straight to their sides and raised to the height of their shoulders. Subjects began to walk forward without shoes after hearing the verbalized cue: “start.” The testers started timing when a participant started to move and stopped the stopwatch when the participant's toes crossed the finish line. This test was performed twice, and the best result was used. Participants could try the test again if they fell from the balance beam in the middle of the test; the testers arranged for an assistant to protect the participants from falling, according to the literature and the timed test involving walking on a balance beam [28–30].

### 2.3. Exercise Intervention

This study used two different protocols involving RPAs as interventions: the experimental group performed novel rhythmic physical activities (NRPA), and the control group performed traditional rhythmic physical activities (TRPA). TRPA exist in kindergartens, which use the form of Chinese radio gymnastics in place. The NRPA were based on the theory of children's dance creation [31] and follow the rules of the motor development of 3 to 5 years old [5], with reference to the curriculum design model of Animal Fun [32] and Animal Tracker [33]. Both RPAs conformed to the nature of children and follow the principle of safety. However, differences between NRPA and TRPA are shown (Table 2). From the design concept, according to the anatomical characteristics of human joints, TRPA focused on the motion of the joints of the body (e.g., head motions and chest motions); NRPA were based on the idea that 3 to 5 years old is a sensitive period of motor development. It emphasized the study of basic motor patterns at this sensitive period. From the design purpose, the TRPA emphasized that children should have daily exercise and promoted physical fitness; NRPA focused on the learning of FMS to achieve the development of physical and mental. In terms of design steps, the TRPA involved determining the music first and then arranging the motions according to the rhythm of the music, and the NRPA determined the motor skills of the exercise of 3- to 5-year-old children in the predesign phase and then combined the determined motor skills with the rhythm and theme of the music (the same music as the TRPA). From the content, the TRPA were mainly based on balance ability completing the flexion, extension, vibration, torsion, and looping of the joints from head to foot, the content was the same for 3 to 5 years old; according to the classification idea of FMS, the NRPA divided the gross motor to be developed in the sensitive period into locomotor, manipulative, and balance ability [1]. Motor parameters (e.g., directions and types) of the NRPA were gradually more complex with age. Examples of NRPA and TRPA are shown in Table 3. From the learning form, the TRPA were single for repetitive practice; the NRPA were variable for repeatedly in a nonrepetitive way. The intervention period was two semesters for one year, and there was a sports class from Monday to Friday for 30 minutes for both groups.

### 2.4. Statistical Analyses

Statistical analyses were performed by using SPSS (Version 22.0). Descriptive statistics were conducted to describe the participants in this study and to calculate the mean and standard deviation. The basic information of the control group and the experimental group was used by independent sample *t*-test. Two-way repeated measures ANOVA was used to compare the influence of groups × time on the related parameters of locomotor, manipulative, and balance ability. Choose Bonferroni's correction when the variance was homogeneous; choose Tukey test for uneven variance. For the parameters with interaction, the paired sample *t*-test was used intergroup, and the independent sample *t*-test was used between the groups. In order to compare the difference of intervention effects, Cohen's d for the difference between two groups in case of quantitative variables was used, and 0.20 is the small effect, 0.50 is the medium effect, and 0.80 is the high effect.

## 3. Results

### 3.1. Locomotor, Manipulative, and Gross Motor Development

Before and after the intervention, the experimental group and the control group were tested by TGMD-2. The scores of each specific motor, locomotor, manipulative, and gross motor development (GMD) are shown in Table 4. Found from the table: before the experiment, there was no significant difference between the experimental group and the control group in scores of the specific motor, locomotor, manipulative, and gross motor at the same ages (*P* > 0.05), showing that the division of the groups was reasonable. Before and after intervention for the control group was compared, except for the scores of the running, leaping, kicking, and underhand rolling of the 3-year-old group and running, galloping, sliding, kicking, striking of the 4-year-old group; -+jumping, catching, throwing, and underhand rolling of the 5-year-old group did not improve significantly (*P* > 0.05), the scores of the galloping and catching of the 3-year-old group and sliding of the 5-year-old group were significantly improved (*P* < 0.05), and the scores of other specific motors, locomotor, manipulative, and GMD were significantly improved (*P* < 0.01). Comparison before and after intervention for the experimental group, except for the scores of the hopping and kicking scores of the 3-year-old group did not improve significantly (*P* > 0.05), the scores of other specific motors, locomotor, manipulative, and GMD were significantly improved (*P* < 0.01). Between the experimental and control groups after the intervention, except for the scores of the jumping, hopping, leaping, and kicking of the 3-year-old group, underhand rolling of the 4-year-old group and hopping of the 5-year-old group had not significantly (*P* > 0.05), the scores of the running, locomotor, dribbling, striking, and underhand rolling of the 3-year-old group, throwing of the 4-year-old group, and leaping and striking of the 5-year-old group were significantly improved (*P* < 0.05); the scores of other specific motors, locomotor, manipulative, and GMD were significantly improved (*P* < 0.01).

### 3.2. Static Balance

The results of the static balance test are shown in Table 5. Comparing the differences between the experimental group and control group, there were no significant differences in the pretest (*P* > 0.05). Comparing the pretest and posttest results within the control group resulted in the following: the group of 3-year-old children, the COP length and distance in the M-L direction during the FEO test, and the COP area and length in the M-L direction during the SFEO test all decreased significantly after the intervention (*P* < 0.05); the COP length and distance in the M-L direction and the COP area during the FEO test the group of 4- and 5-year-old children, the COP distance in the A-P direction during the SFEO test the group of 4-year-old children, and the COP area during the SFEO test the group of 5-year-old children all showed a significant decrease after the intervention (*P* < 0.05). Comparing the pretest with the posttest results within the experimental group resulted in the following: the group of 3-year-old children, the COP length during the FEO test; the COP length, area and distance in the M-L direction during the FEC test; and the COP length, area, and distance in the A-P and M-L directions during the SFEO test, all decreased significantly after the intervention (*P* < 0.05), while the COP area decreased significantly (*P* < 0.01). The group of 4-year-old children, the COP length, area, and distance in the A-P and M-L directions during the static balance test all decreased significantly after the intervention (*P* < 0.05), and the COP area during the FEO and FEC tests and the COP length and distance in the M-L direction during the SFEO test, all showed a significant decrease (*P* < 0.01). The group of 5-year-old children, the COP length, area, and distance in the M-L direction all decreased significantly after the intervention (*P* < 0.05). Between the experimental and control groups after the intervention, the group of 3-year-old children, the COP length during the FEO test, the COP length and area during the FEC test, and the COP area and distance in the A-P and M-L directions during the SFEO test all decreased significantly (*P* < 0.05); the group of 4-year-old children and the COP length, area, and distance in the A-P direction on the static balance test all decreased significantly (*P* < 0.05); the group of 5-year-old children and the COP length and distance in the A-P direction during the SFEO test decreased significantly (*P* < 0.05); area during the SFEO test decreased significantly (*P* < 0.01).

### 3.3. Dynamic Balance

The results for dynamic balance test are shown in Table 6. Both the experimental group and control group had no significant differences in pretest results (*P* > 0.05). Comparing the pretest results with the posttest results within the control group resulted in the following: the group of 3-, 4-, and 5-year-old children, the measures all decreased significantly after the intervention (*P* < 0.05). Comparing the pretest results with the posttest results within the experimental group resulted in the following: the group of 3-year-old children showed a significant decline in walking time on a balance beam (*P* < 0.05), and the group of 4- and 5-year-old children showed significant declines in walking time on a balance beam (*P* < 0.01). Between the experimental and control groups after the intervention, the group of 3-year-old children showed a significant decline (*P* < 0.05), and the group of 4- and 5-year-old children showed a significant decline (*P* < 0.01).

## 4. Discussion

TGMD-2 can evaluate the competence of locomotor and manipulative, standing on Tekscan and walking on balance beam can evaluate the stability competence of static and dynamic; all of them can assess FMS. Among the 7 locomotor and 6 manipulative, the hopping and kicking of the 3-year-old group were not improved, and other motor skills as well as the total scores of locomotor, manipulative, and gross motor had all been improved due to the intervention of the NRPA course. This was not surprising given that the children had opportunity to practice structured program. Before and after the intervention, hopping and kicking improved the least, as hopping is a complex skill requiring a considerable degree of strength and coordination, and it is a later maturing skill [34]. With respect to kicking, performance would not be expected in 3- to 5-year-old children [34]. In the static balance test, under three standing conditions, the children improved at least one index in all indexes, and the dynamic balance ability was improved for 3- to 5-year-old children. Furthermore, NRPA had significantly higher posttest scores than TRPA.

Physical activities with imagination are more suitable for children by music [35]. Animal Fun [32] and Animal Tracker [33] are courses that imitate various animal movements with music, and the results proved that the two program helping children learn motor skills and had a positive effect on the development of children's multiple intelligences [36]. NRPA were based on the principles of children's dance creation, which were the character's life, the imitation of the action and the gameplay [31], and followed the laws of the children's motor development including locomotor, manipulative, and nonlocomotor skills. With age increasing and music theme changing, the movement parameters could be changed, so children learned in an incompletely repetitive environment. Schema Theory holds that the process of motor learning is the process of the model of motor establishing. The more changing in movement parameters, the more helpful to establish the best basic model and achieve the best learning effect [37]. For example, in order to learn the movement pattern of “walking,” children can practice “walking” in different directions, different postures, and different speeds. From the perspective of practice methods, Moore et al. [38] believed that learning a new motor skill like remembering a thing, the process must be repeated over and over, so repeated practice was necessary to learn motor skills. However, the Forgetting Hypothesis believed that nonrepetitive practice would make people forget, but the process of regenerating the motor is the process of reinforcement learning [39]. Lee et al. [40] also proposed that appropriate practice is not to practice repeatedly in a fixed form. Therefore, the effect of incomplete repetitive exercises is better than repetitive exercises. In addition, Sacha & Russ [35] have found that certain changes of dance content could enhance children's motivation and interest in learning. Therefore, NRPA could not only stimulate children's motivation to practice FMS but also cultivate children's appreciation of music and dance and promote the coordinated development of children's physical and mind.

A limitation of this study was the lack of follow-up procedures. It still remains to be determined if the skill improvements gained from NRPA were maintained over time. Future research should attempt to longitudinally track children receiving such program.

## 5. Conclusion

NRPA, which were arranged according to the concept of the sensitive period of motor development, could effectively promote the improvement of children's FMS due to the variability of movement parameters and the incomplete repeatability of practice methods. Therefore, NRPA can be widely promoted in Chinese kindergartens or other countries.

## Figures and Tables

**Table 1:** Basic information about the physical conditions of the participants.

Test	Age (years old)	Experimental group				Control group			
		Number	Height (cm)	Weight (kg)	BMI (kg/m^2^)	Number	Height (cm)	Weight (kg)	BMI (kg/m^2^)
TGMD-2	3	52	100.51 ± 4.33	16.34 ± 2.61	16.32 ± 2.22	56	98.28 ± 3.42	15.17 ± 1.62	15.66 ± 1.16
	4	67	106.10 ± 5.00	17.41 ± 2.13	15.45 ± 0.98	64	106.20 ± 5.06	17.35 ± 2.22	15.40 ± 1.30
	5	23	111.13 ± 3.91	19.13 ± 2.51	16.51 ± 1.32	27	113.10 ± 4.46	20.02 ± 2.79	16.60 ± 1.53
Balance	3	11	101.32 ± 3.57	16.59 ± 2.06	16.16 ± 1.45	11	97.45 ± 4.97	15.08 ± 2.32	15.88 ± 0.98
	4	11	108.58 ± 3.47	18.31 ± 1.92	15.55 ± 0.93	11	109.19 ± 5.18	18.10 ± 1.78	15.19 ± 1.20
	5	8	113.75 ± 1.77	21.00 ± 2.12	16.23 ± 1.35	8	115.92 ± 1.85	21.00 ± 2.12	16.63 ± 1.22

Notes. For children of the same age, no significant differences in physical condition were found between the experimental group and the control group (*P* > 0.05).

**Table 2:** Differences between NRPA and TRPA.

	The control group (TRPA)	The experimental group (NRPA)
Concept	According to anatomical characteristics such as within the extent of motion of the joints of the body	According to sensitive period of motor development
Purpose	Ensuring daily exercise and promoting children's physical fitness	Learning fundamental movement skills, developing physical health and mental health
Procedure	Designing motions based on the rhythm of music	Combining the determined motions with the rhythm and theme of the music
Content	In-place stability from head to foot, no age difference	Locomotor, manipulative, and stability to be developed in the sensitive period, age difference
Learning form	Single, repeated practice	Variable, not exactly repeating

Note. NRPA: novel rhythmic physical activities; TRPA: traditional rhythmic physical activities.

**Table 3:** Movement examples of NRPA and TRPA.

Term	Song	Movement of NRPA	NSAR (years)	Movement of TRPA	TSAR (years)	Dur (s)	Tempo (beat/min)
First	Hit the gyro on the ice	B: flexing, extending, turning L: squatting, walking M: throwing, hitting	3	With the rhythm of the same music, the flexion, extension, vibration, torsion, and looping of the joints are completed from head to foot in place.	No age differences	145	132
	Pony crossing the river	B: standing on a single leg, flexing, extending, turning L: walking, galloping, sliding M: rolling	4			156	130
	White dragon horse	B: standing on the tiptoes, standing on a single leg, rotating, swaying L: walking backwards, running, skipping, galloping M: holding, throwing, hitting	5			170	140
Second	Where does dad go	B: dropping, lifting, flexing, extending, turning L: walking, jumping M: kicking	3			156	122
	My chick	B: standing on a single leg, flexing, extending, flapping, rotating L: walking, running, leaping M: catching, throwing	4			192	132
	Be your good friend	B: standing on the tiptoes, standing on a single leg, flexing, extending, rotating L: walking sideways, running, sliding, hopping M: holding, dribbling, catching	5			227	140

Note. B: balance; L: locomotor; M: manipulative; NRPA: novel rhythmic physical activities; TRPA: traditional rhythmic physical activities; NSAR: novel suitable age range; TSAR: traditional suitable age range.

**Table 4:** Scores from the TGMD-2 for Two Groups before and after the Intervention (unit: score).

Group		3 years		4 years		5 years	
		Control group (*n* = 56)	Experimental group (*n* = 52)	Control group (*n* = 64)	Experimental group (*n* = 67)	Control group (*n* = 27)	Experimental group (*n* = 23)
Running	Pretest	5.42 ± 0.88	5.54 ± 0.56	6.03 ± 0.88	6.15 ± 0.88	7.23 ± 0.65	7.95 ± 0.83
	Posttest	5.70 ± 0.91	6.11 ± 0.74^4)5)^	6.17 ± 0.78	6.99 ± 0.86^4)6)^	7.52 ± 0.74^2)^	8.55 ± 0.86^4)6)^
	*Δ*	0.62 ± 0.03	0.57 ± 0.05	0.14 ± 0.03	0.84 ± 0.07	0.29 ± 0.01	0.60 ± 0.03
	Cohen's d	0.28	0.87	0.17	0.97	0.42	0.90
Horizontal jumping	Pretest	3.88 ± 1.50	3.82 ± 1.36	5.02 ± 1.64	4.94 ± 1.52	6.31 ± 1.24	6.52 ± 1.13
	Posttest	4.35 ± 1.62^2)^	4.97 ± 1.73^4)^	5.60 ± 1.71^2)^	6.29 ± 1.92^4)6)^	6.50 ± 1.34	7.31 ± 1.24^4)6)^
	*Δ*	0.47 ± 0.12	1.15 ± 0.37	0.58 ± 0.07	1.35 ± 0.40	0.19 ± 0.10	0.79 ± 0.11
	Cohen's d	0.30	0.73	0.41	0.77	0.14	0.66
Leaping	Pretest	2.42 ± 1.16	2.65 ± 1.83	4.57 ± 1.84	4.21 ± 1.80	6.04 ± 1.18	6.09 ± 1.27
	Posttest	4.05 ± 1.63^2)^	4.59 ± 2.52^4)^	5.53 ± 1.92^2)^	5.84 ± 2.10^4)6)^	6.92 ± 1.19^2)^	7.24 ± 1.39^4)5)^
	*Δ*	1.63 ± 0.47	1.94 ± 0.69	0.96 ± 0.08	1.63 ± 0.30	0.88 ± 0.01	1.15 ± 0.12
	Cohen's d	0.39	0.88	0.61	0.83	0.77	0.97
Hopping	Pretest	3.71 ± 1.10	3.77 ± 1.08	3.81 ± 0.82	3.93 ± 1.07	4.27 ± 1.22	4.77 ± 0.92
	Posttest	3.91 ± 1.38	4.06 ± 1.30	4.31 ± 1.36^2)^	4.65 ± 0.68^4)6)^	5.15 ± 1.21^2)^	5.45 ± 0.60^4)^
	*Δ*	0.20 ± 0.28	0.29 ± 0.22	0.53 ± 0.54	0.52 ± 0.09	0.88 ± 0.01	0.68 ± 0.32
	Cohen's d	0.16	0.24	0.44	0.80	0.72	0.87
Galloping	Pretest	3.14 ± 1.10	3.84 ± 1.83	4.86 ± 1.26	4.57 ± 1.63	5.38 ± 1.81	5.73 ± 1.49
	Posttest	3.79 ± 1.34^1)^	4.73 ± 1.12^4)6)^	5.06 ± 1.36	5.61 ± 1.08^4)6)^	6.69 ± 1.50^2)^	6.93 ± 0.94^4)6)^
	*Δ*	0.65 ± 0.24	0.89 ± 0.71	0.20 ± 0.30	1.04 ± 0.55	1.31 ± 0.31	1.20 ± 0.55
	Cohen's d	0.36	0.59	0.15	0.75	0.17	0.96
Sliding	Pretest	3.93 ± 2.17	3.84 ± 1.94	5.74 ± 1.19	5.52 ± 1.48	7.27 ± 1.02	6.95 ± 1.30
	Posttest	5.12 ± 1.52^2)^	5.86 ± 2.19^4)6)^	5.95 ± 0.93	6.59 ± 0.96^4)6)^	7.96 ± 0.82^1)^	7.97 ± 0.67^4)6)^
	*Δ*	1.19 ± 0.65	2.02 ± 0.25	0.21 ± 0.26	1.07 ± 0.52	0.69 ± 0.20	1.02 ± 0.62
	Cohen's d	0.63	0.97	0.19	0.86	0.73	0.98
Locomotor score	Pretest	21.63 ± 6.35	22.05 ± 6.09	30.05 ± 5.20	29.23 ± 5.26	33.50 ± 6.67	33.27 ± 6.09
	Posttest	26.26 ± 6.05^2)^	27.27 ± 4.60^4)5)^	32.33 ± 4.32^2)^	33.97 ± 4.84^4)6)^	37.92 ± 6.30^2)^	39.27 ± 6.71^4)6)^
	*Δ*	4.63 ± 0.20	5.22 ± 1.49	2.28 ± 0.81	4.74 ± 0.42	4.42 ± 0.37	6.00 ± 0.62
	Cohen's d	0.74	0.96	0.89	0.93	0.68	0.94
Dribbling	Pretest	1.00 ± 0.77	0.91 ± 0.70	2.24 ± 1.90	3.11 ± 1.80	5.19 ± 1.36	5.15 ± 1.25
	Posttest	1.56 ± 0.98^2)^	1.68 ± 0.93^4)5)^	3.33 ± 1.53^2)^	4.72 ± 1.73^4)6)^	6.08 ± 1.02^2)^	6.24 ± 1.26^4)6)^
	*Δ*	0.56 ± 0.21	0.73 ± 0.23	1.09 ± 0.63	1.61 ± 0.07	0.89 ± 0.34	1.09 ± 0.01
	Cohen's d	0.63	0.93	0.63	0.91	0.74	0.86
Kicking	Pretest	4.70 ± 1.28	4.87 ± 1.14	5.19 ± 1.02	5.21 ± 0.97	6.31 ± 1.19	6.59 ± 1.10
	Posttest	5.07 ± 1.12	5.27 ± 1.01	5.52 ± 1.03	6.18 ± 0.86^4)6)^	7.35 ± 1.55^2)^	7.95 ± 1.90^4)5)^
	*Δ*	0.37 ± 0.16	0.40 ± 0.13	0.33 ± 0.01	0.97 ± 0.11	1.04 ± 0.36	1.36 ± 0.80
	Cohen's d	0.30	0.37	0.32	0.86	0.75	0.87
Catching	Pretest	1.84 ± 1.15	1.89 ± 0.69	2.19 ± 1.21	2.61 ± 1.10	3.65 ± 1.33	3.64 ± 1.33
	Posttest	2.26 ± 1.20^1)^	2.49 ± 0.88^4)6)^	2.53 ± 0.99^2)^	3.26 ± 0.83^4)6)^	3.95 ± 1.32	4.55 ± 0.85^4)6)^
	*Δ*	0.42 ± 0.05	0.60 ± 0.19	0.33 ± 0.12	0.55 ± 0.27	0.30 ± 0.01	0.91 ± 0.48
	Cohen's d	0.40	0.75	0.30	0.65	0.23	0.86
Striking	Pretest	4.00 ± 1.69	4.14 ± 1.56	5.07 ± 1.75	5.03 ± 1.62	5.28 ± 1.94	5.25 ± 2.01
	Posttest	5.16 ± 1.73^2)^	5.44 ± 1.91^4)5)^	5.54 ± 1.73	6.13 ± 1.42^4)6)^	6.62 ± 1.53^2)^	7.11 ± 2.44^4)5)^
	*Δ*	1.16 ± 0.04	1.30 ± 0.35	0.47 ± 0.04	1.10 ± 0.20	1.34 ± 0.41	1.84 ± 0.43
	Cohen's d	0.67	0.74	0.27	0.72	0.76	0.83
Throwing	Pretest	2.14 ± 0.86	2.30 ± 1.05	2.78 ± 1.36	2.72 ± 1.16	3.85 ± 1.38	3.82 ± 1.45
	Posttest	2.51 ± 1.22^2)^	3.30 ± 1.18^4)6)^	3.57 ± 1.01^2)^	3.97 ± 1.25^4)5)^	4.06 ± 1.27	5.41 ± 1.31^4)6)^
	*Δ*	0.37 ± 0.26	1.00 ± 0.13	0.79 ± 0.24	1.25 ± 0.09	0.21 ± 0.11	0.59 ± 0.14
	Cohen's d	0.35	0.89	0.65	0.90	0.17	0.74
Underhand rolling	Pretest	3.67 ± 0.92	3.70 ± 0.70	3.95 ± 0.66	3.94 ± 1.08	4.65 ± 1.38	4.21 ± 1.34
	Posttest	3.93 ± 0.89	4.27 ± 0.77^4)5)^	4.34 ± 0.91^2)^	4.87 ± 1.26^4)^	4.92 ± 1.06	5.04 ± 1.13^4)6)^
	*Δ*	0.74 ± 0.03	0.57 ± 0.07	0.39 ± 0.25	0.93 ± 0.18	0.37 ± 0.32	0.83 ± 0.21
	Cohen's d	0.28	0.77	0.49	0.79	0.21	0.66
Manipulative score	Pretest	17.12 ± 4.67	17.51 ± 3.76	21.41 ± 4.14	21.72 ± 4.02	27.73 ± 5.42	27.95 ± 5.64
	Posttest	20.49 ± 5.20^2)^	20.86 ± 4.23^4)6)^	23.53 ± 4.04^2)^	25.04 ± 3.36^4)6)^	30.38 ± 3.73^2)^	31.86 ± 3.73^4)6)^
	*Δ*	3.37 ± 0.53	3.35 ± 0.47	2.12 ± 0.10	3.32 ± 0.66	2.65 ± 1.69	4.91 ± 1.91
	Cohen's d	0.68	0.83	0.51	0.89	0.56	0.81
Scores of GM	Pretest	38.74 ± 10.28	39.57 ± 9.67	51.47 ± 18.22	51.23 ± 17.29	55.95 ± 17.47	61.23 ± 17.29
	Posttest	42.74 ± 10.15^2)^	47.14 ± 10.84^4)6)^	58.86 ± 17.02^2)^	62.39 ± 16.47^4)6)^	65.31 ± 13.40^2)^	75.14 ± 14.19^4)6)^
	*Δ*	4.00 ± 0.13	7.57 ± 3.17	7.39 ± 1.02	4.08 ± 1.85	9.36 ± 4.07	14.91 ± 3.10
	Cohen's d	0.39	0.73	0.41	0.66	0.60	0.87

Note. Comparison of the control group before and after the intervention: ^1)^ = *P* < 0.05, ^2)^ = P < 0.01. Comparison of the experimental group before and after the intervention; ^3)^ = *P* < 0.05, ^4)^ = *P* < 0.01. Comparison of the experimental group and control group after the intervention; ^5)^ = *P* < 0.05, ^6)^ = *P* < 0.01. *Δ* = mean and standard deviation of the change from pre- to postintervention (the above is the same below).

**Table 5:** Results from the static balance test for two groups before and after the intervention.

Group	Index		3 years		4 years		5 years	
			Control group (*n* = 22)	Experimental group (*n* = 22)	Control group (*n* = 16)	Experimental group (*n* = 22)	Control group (*n* = 22)	Experimental group (*n* = 16)
FEO	Area	Pretest	4.96 ± 2.86	4.84 ± 3.71	4.25 ± 2.18	4.17 ± 1.79	3.06 ± 2.01	2.94 ± 1.72
		Posttest	4.16 ± 2.66	3.49 ± 2.53	3.28 ± 2.89^1)^	3.24 ± 1.64^4)5)^	2.16 ± 1.94^1)^	1.86 ± 0.78^3)5)^
		*Δ*	−0.80 ± 0.20	−1.35 ± 1.19	−0.97 ± 0.18	−0.96 ± 0.01	−1.08 ± 0.06	−1.15 ± 0.02
		Cohen's d	0.28	0.42	0.37	0.62	0.45	0.80
	Length	Pretest	108.09 ± 68.22	105.30 ± 51.06	75.37 ± 29.96	73.33 ± 25.88	43.10 ± 16.72	43.37 ± 17.20
		Posttest	92.1 ± 38.22	69.84 ± 39.57^3)5)^	63.18 ± 27.96^1)^	56.66 ± 24.06^3)5)^	38.92 ± 15.71	35.53 ± 12.01^3)5)^
		*Δ*	−16.99 ± 5.22	−35.46 ± 6.31	−12.19 ± 2.13	−16.67 ± 2.37	−4.18 ± 1.01	−7.84 ± 5.19
		Cohen's d	0.28	0.77	0.40	0.81	0.25	0.57
	A-P	Pretest	3.12 ± 1.13	2.91 ± 1.11	2.58 ± 0.85	2.61 ± 1.04	1.97 ± 0.85	1.96 ± 0.54
		Posttest	2.89 ± 0.92	2.60 ± 0.99	2.31 ± 0.99	2.03 ± 0.91^3)5)^	1.89 ± 0.72	1.84 ± 0.50
		*Δ*	−0.28 ± 0.02	−0.34 ± 0.01	−0.27 ± 0.01	−0.58 ± 0.03	−0.08 ± 0.01	−0.32 ± 0.04
		Cohen's d	0.22	0.29	0.29	0.59	0.10	0.23
	M-L	Pretest	4.19 ± 2.16	4.24 ± 2.44	3.63 ± 1.85	3.59 ± 2.42	2.63 ± 1.53	2.59 ± 1.62
		Posttest	3.64 ± 1.98	3.57 ± 1.96	2.53 ± 1.56^1)^	2.16 ± 1.82^3)^	2.16 ± 0.82^1)^	1.96 ± 0.87^3)^
		*Δ*	−0.55 ± 0.07	−0.67 ± 0.08	−1.15 ± 0.09	−1.43 ± 0.60	−0.47 ± 0.09	−0.63 ± 0.04
		Cohen's d	0.26	0.30	0.64	0.66	0.38	0.48
**FEC**	Area	Pretest	5.11 ± 1.68	5.10 ± 2.84	4.76 ± 1.97	4.78 ± 1.84	3.39 ± 1.56	3.24 ± 1.53
		Posttest	4.71 ± 1.54	3.64 ± 2.26^3)5)^	4.20 ± 1.76	3.96 ± 1.62^4)5)^	2.98 ± 1.32	2.46 ± 1.36^3)5)^
		*Δ*	−0.40 ± 0.14	−1.46 ± 0.58	−0.56 ± 0.21	−0.82 ± 0.22	−0.41 ± 0.34	−0.78 ± 0.17
		Cohen's d	0.24	0.56	0.29	0.47	0.28	0.53
	Length	Pretest	113.51 ± 38.03	110.17 ± 43.17	85.32 ± 27.59	85.53 ± 26.61	55.09 ± 19.90	53.75 ± 12.81
		Posttest	88.32 ± 30.11^1)^	78.57 ± 29.34^3)5)^	68.20 ± 20.74^1)^	64.52 ± 20.97^3)^	45.42 ± 15.88^1)^	42.79 ± 10.34^3)5)^
		*Δ*	−25.19 ± 8.13	−31.63 ± 14.23	−17.12 ± 6.85	−21.01 ± 5.64	−9.67 ± 4.02	−10.04 ± 2.47
		Cohen's d	0.73	0.85	0.70	0.87	0.53	0.94
	A-P	Pretest	4.19 ± 1.34	4.02 ± 1.59	3.69 ± 1.12	3.67 ± 1.13	3.12 ± 0.88	3.09 ± 0.86
		Posttest	3.92 ± 1.11	3.69 ± 1.13	3.11 ± 0.99^1)^	2.89 ± 0.78^3)5)^	2.89 ± 0.78	2.48 ± 0.46
		*Δ*	−0.27 ± 0.23	−0.33 ± 0.46	−0.58 ± 0.1	−0.78 ± ±0.35	−0.23 ± 0.10	−0.61 ± 0.40
		Cohen's d	0.21	0.23	0.54	0.80	0.27	0.88
	M-L	Pretest	5.25 ± 2.14	5.22 ± 1.84	4.48 ± 1.11	4.47 ± 0.93	3.81 ± 0.69	3.86 ± 0.88
		Posttest	4.31 ± 1.78^1)^	4.12 ± 1.43^3)^	3.94 ± 1.02^1)^	3.76 ± 1.03^3)^	3.49 ± 0.50^1)^	3.29 ± 0.75^3)^
		*Δ*	−0.94 ± 0.36	−1.10 ± 0.41	−0.54 ± 0.09	−0.71 ± 0.90	−0.32 ± 0.19	−0.57 ± 0.13
		Cohen's d	0.47	0.66	0.50	0.72	0.53	0.75
SFEO	Area	Pretest	20.71 ± 8.03	20.56 ± 8.54	17.98 ± 7.40	18.11 ± 7.67	12.81 ± 4.59	12.71 ± 4.88
		Posttest	17.73 ± 5.68^1)^	15.66 ± 5.16^4)5)^	15.95 ± 6.23	14.97 ± 5.58^3)6)^	10.41 ± 3.03^1)^	9.52 ± 3.79^3)5)^
		*Δ*	−2.98 ± 0.35	−4.90 ± 2.38	−2.03 ± 1.17	−3.14 ± 1.05	−2.40 ± 1.56	−3.19 ± 1.09
		Cohen's d	0.42	0.69	0.28	0.46	0.61	0.73
	Length	Pretest	123.15 ± 26.41	112.06 ± 28.06	98.33 ± 31.45	99.85 ± 26.10	70.45 ± 13.34	69.06 ± 13.92
		Posttest	105.76 ± 23.79^1)^	103.17 ± 22.46^3)^	87.38 ± 21.25^1)^	85.73 ± 15.42^4)5)^	63.05 ± 7.69^1)^	59.38 ± 8.62^3)5)^
		*Δ*	−18.39 ± 3.41	−18.89 ± 5.60	−10.95 ± 4.25	−14.12 ± 3.07	−7.45 ± 2.16	−9.68 ± 2.27
		Cohen's d	0.69	0.74	0.40	0.65	0.67	0.83
	A-P	Pretest	9.39 ± 4.05	9.44 ± 4.13	5.38 ± 2.04	5.34 ± 1.48	4.54 ± 1.09	4.55 ± 1.11
		Posttest	8.29 ± 3.62	8.22 ± 3.45^3)5)^	4.88 ± 1.94	4.43 ± 1.23^3)^	4.39 ± 1.02	4.29 ± 1.00
		*Δ*	−1.10 ± 0.43	−1.22 ± 0.26	−0.50 ± 0.10	−0.81 ± 0.25	−0.15 ± 0.07	−0.26 ± 0.11
		Cohen's d	0.28	0.32	0.25	0.66	0.14	0.24
	M-L	Pretest	9.27 ± 2.81	9.11 ± 2.59	5.85 ± 1.80	5.72 ± 1.84	4.72 ± 1.11	4.52 ± 1.83
		Posttest	7.27 ± 2.31^1)^	7.04 ± 2.23^3)5)^	4.87 ± 1.26^1)^	4.30 ± 1.72^4)5)^	4.28 ± 1.01^1)^	3.22 ± 1.77^3)5)^
		*Δ*	−2.00 ± 0.50	−2.07 ± 0.36	−0.982 ± 0.54	−1.42 ± 0.12	−0.44 ± 0.10	−1.30 ± 0.06
		Cohen's d	0.77	0.85	0.63	0.79	0.41	0.72

*Note.* FEO: both feet with eyes open; FEC: both feet with eyes closed; SFEO: a single foot with eyes open; A-P: anterior-posterior; M-L: medial-lateral. Units: area: cm^2^; length: cm; A-P: cm; M-L: cm.

**Table 6:** Results from the dynamic balance test for two groups before and after the intervention (unit: s).

Group	3 years		4 years		5 years	
	Control group (*n* = 22)	Experimental group (*n* = 22)	Control group (*n* = 16)	Experimental group (*n* = 22)	Control group (*n* = 22)	Experimental group (*n* = 16)
Pretest	15.87 ± 3.29	15.67 ± 3.23	12.31 ± 2.09	12.71 ± 2.08	8.75 ± 1.23	8.61 ± 1.08
Posttest	13.87 ± 3.10^1)^	12.87 ± 2.96^4)5)^	11.24 ± 2.37^1)^	10.94 ± 1.92^4)6)^	8.29 ± 1.22^1)^	7.63 ± 1.16^4)6)^
*Δ*	−2.00 ± 0.19	−2.80 ± 0.27	−1.07 ± 0.18	−1.77 ± 0.16	−0.46 ± 0.01	−0.94 ± 0.12
Cohen's d	0.62	0.90	0.47	0.88	0.37	0.80

## References

[1] Gabbard C. (2018). *Lifelong Motor Development*.

[2] Metcalfe J., Clark J. E. (2002). The mountain of motor development: a metaphor. Motor development: research and reviews. *Reston (VA): National Association of Sport & Physical Education*.

[3] Robinson L. E., Stodden D. F., Barnett L. M. (2015). Motor competence and its effect on positive developmental trajectories of health. *Sports Medicine*.

[4] Cools W., De Martelaer K., Samaey C., Andries C. (2014). Movement skill assessment of typically developing preschool children: a review of seven movement skill assessment tools. *Journal of Sports Science & Medicine*.

[5] Gallahue D. L., Ozmun J. C. (2011). *Understanding Motor Development: Infants, Children, Adolescents, Adults*.

[6] Stodden D. F., True L. K., Langendorfer S. J., Gao Z. (2013). Associations among selected motor skills and health-related fitness: indirect evidence for Seefeldt’s proficiency barrier in young adults?. *Research Quarterly for Exercise and Sport*.

[7] Cliff D. P., Okely A. D., Morgan P. J., Jones R. A., Steele J. R., Baur L. A. (2012). Proficiency deficiency: mastery of fundamental movement skills and skill components in overweight and obese children. *Obesity*.

[8] Newell K., Thomas J. (1984). Physical constraints to development of motor skills. *Motor Development during Childhood and Adolescence*.

[9] Hamilton M., Liu T. (2018). The effects of an intervention on the gross and fine motor skills of hispanic Pre-K children from low SES backgrounds. *Early Childhood Education Journal*.

[10] Clark J. E., Clements R. L., Guddemi M. (2009). *Active Start: A Statement of Physical Activity Guidelines for Children Birth to Five Years*.

[11] Macdonald D. (2013). The new Australian Health and Physical Education Curriculum: a case of/for gradualism in curriculum reform. *Asia-Pacific Journal of Health Sport and Physical Education*.

[12] Hardy L. L., King L., Farrell L., Macniven R., Howlett S. (2010). Fundamental movement skills among Australian preschool children. *Journal of Science and Medicine in Sport*.

[13] Chatzihidiroglou P., Chatzopoulos D., Lykesas G., Doganis G. (2018). Dancing effects on preschoolers’ sensorimotor synchronization, balance, and movement reaction time. *Perceptual & Motor Skills*.

[14] Qin D. X. (1987). *Introduction of Chinese and Foreign Music Teaching Methods*.

[15] Jaques-Dalcroze E. (1930). *Eurhythmics Art and Education*.

[16] Ulrich D. A. (2000). *Test of Gross Motor Development (Second Edition) Examiner’s Manual*.

[17] Westendorp M., Hartman E., Houwen S., Smith J., Visscher C. (2011). The relationship between gross motor skills and academic achievement in children with learning disabilities. *Research in Developmental Disabilities*.

[18] Li J., Ma H. X. (2007). Study of the credibility and validity of the test of gross motor development of children. *Journal of Physical Education*.

[19] Pedersen L. K., Martinkevich P., Ege S. (2016). Postural seated balance in children can be assessed with good reliability. *Gait & Posture*.

[20] Coda A., Carline T., Santos D. (2014). Repeatability and reproducibility of the Tekscan HR-Walkway system in healthy children. *The Foot*.

[21] Cooper A., Chhina H., Howren A., Alvarez C. (2014). The contralateral foot in children with unilateral clubfoot, is the unaffected side normal?. *Gait & Posture*.

[22] Cousins S. D., Morrison S. C., Drechsler W. I. (2012). The reliability of plantar pressure assessment during barefoot level walking in children aged 7-11 years. *Journal of Foot and Ankle Research*.

[23] Brenton-Rule A., Mattock J., Carroll M. (2012). Reliability of the TekScan MatScan® system for the measurement of postural stability in older people with rheumatoid arthritis. *Journal Foot Ankle Research*.

[24] Houston M. N., Peck K. Y., Malvasi S. R., Roach S. P., Svoboda S. J., Cameron K. L. (2019). Reference values for the Balance Error Scoring System as measured by the Tekscan MobileMat™ in a physically active population. *Brain Injury*.

[25] Winter D. A. (2009). *Biomechanics and Motor Control of Human Movements*.

[26] Fu L. M., Cui J. H., Feng J. T. (2015). Comparative study and construction of evaluating model on the static balance of children aged 6-8. *Journal Tianjin University of sport*.

[27] Liu Y. (2007). Development of balance measurement and training studies for human beings. *Journal Shenyang Sport University*.

[28] Arevalo-Mora J. F., Reina-Bueno M., Munuera P. V. (2016). Influence of children’s foot type on their physical motor performance. *Journal of the American Podiatric Medical Association*.

[29] Coetzee D. (2016). Strength, running speed, agility and balance profiles of nine to 10-year-old learners in the North West Province of South Africa: the NW-child study. *South African Journal for Research in Sport, Physical Education and Recreation*.

[30] Kakebeeke T. H., Locatell I., Rousson V., Caflisch I., Jenni O. G. (2012). Improvement in gross motor performance between 3 and 5 years of age. *Perceptual and Motor Skills*.

[31] Jia R., L. (2007). *Dance Creation Tutorial*.

[32] Piek J. P., McLaren S., Kane R. (2013). Does the Animal Fun program improve motor performance in children aged 4-6 years?. *Human Movement Science*.

[33] Williams C. L., Carter B. J., Kibbe D. L., Dennison D. (2009). Increasing physical activity in preschool: a pilot study to evaluate animal trackers. *Journal of Nutrition Education and Behavior*.

[34] Payne V. G., Isaacs L. D. (2002). *Human Motor Development: A Lifespan Approach*.

[35] Sacha T. J., Russ S. W. (2006). Effects of pretend imagery on learning dance in preschool children. *Early Childhood Education Journal*.

[36] Su Y. T. (2006). *The Development, Implementation, and Interpretation of children’s Rhythmic Activities Using the Theory of Multiple Intelligences*.

[37] Schmidt R. A., Timothy D. L. (2010). *Motor Control and Learning*.

[38] Moore J. B., Reeve T. G., Pissanos B. (1981). Effects of variability of practice in a movement education program on motor skill performance. *Perceptual and Motor Skill*.

[39] Cuddy L. J., Jacoby L. L. (1982). When forgetting helps memory: an analysis of repetition effects. *Journal of Verbal Learning and Verbal Behavior*.

[40] Lee T. D., Swanson L. R., Hall A. L. (1991). What is repeated in a repetition? Effects of practice conditions on motor skill acquisition. *Physical Therapy*.

